# Frontotemporal dementias: Recent advances and current controversies

**DOI:** 10.4103/0972-2327.74249

**Published:** 2010-12

**Authors:** Cristian E. Leyton, John R. Hodges

**Affiliations:** Neuroscience Research Australia (NeuRA), The University of New South Wales, Sydney, Australia

**Keywords:** Frontotemporal dementia, progressive nonfluent aphasia, semantic dementia, taupathies, TDP-43

## Abstract

Frontotemporal dementia (FTD) syndromes comprise a heterogeneous group of neurodegenerative conditions characterized by atrophy in the frontal and temporal lobes. Three main clinical variants are recognized: Behavioral variant (bv-FTD), Semantic dementia (SD), and Progressive nonfluent aphasia (PNFA). However, logopenic/phonological (LPA) variant has been recently described, showing a distinctive pattern of brain atrophy and often associated to Alzheimer’s disease pathology. The diagnosis of FTD is challenging, since there is clinical, pathological, and genetic overlap between the variants and other neurodegenerative diseases, such as motoneuron disease (MND) and corticobasal degeneration (CBD). In addition, patients with gene mutations (tau and progranulin) display an inconsistent clinical phenotype and the correspondence between the clinical variant and its pathology is unpredictable. New cognitive tests based on social cognition and emotional recognition together with advances in molecular pathology and genetics have contributed to an improved understanding. There is now a real possibility of accurate biomarkers for early diagnosis. The present review concentrates on new insights and debates in FTD.

## Introduction

Frontotemporal dementia (FTD) encompasses a group of neurodegenerative diseases characterized by focal atrophy of frontal and anterior temporal lobes and non-Alzheimer pathology.[1,2] In people under 65 years of age, FTD is as common as Alzheimer’s disease (AD) and its prevalence has been estimated in 15 per 100,000 patients between 45 to 64 years of age.[3]

Patients with FTD display a heterogeneous clinical picture, which may include behavioral, cognitive, and motor manifestations.[4,5] However, based on the predominant initial symptoms, FTD can be readily separated into two groups: the behavioral variant (bv -FTD), which is characterized by loss of insight, personality changes, and disturbances in social cognition[1] and the language variant, also referred as primary progressive aphasia (PPA).[6] The latter can be further divided into a well-defined clinical-pathological entity, semantic dementia (SD),[7,8] and progressive nonfluent aphasia (PNFA).[1,8–11]

Despite this classification, there is a clinical, pathological, and genetic overlap. For instance, SD cases may develop features of bv-FTD,[12] and patients with the clinical variant often have common areas of brain atrophy[13] and family history of another variant.[14] Moreover, there is increasing evidence of overlap between FTD and other neurodegenerative disease, notably Motor Neuron disease (MND),[15] Progressive Supranuclear Palsy (PSP), and Corticobasal degeneration (CBD).[16,17] For example, cases initially diagnosed as PNFA may end up showing a clinical picture and pathology of CBD.[18] Indeed, some argue that those entities should all be included under the rubric of Pick’s complex.[19]

Differentiating one variant of FTD from another, as well as from other neurodegenerative and nondegenerative diseases (particularly psychiatric conditions) remains challenging.[20] Fortunately, recent advances in molecular pathology and genetics, improved imaging techniques, and better clinical descriptions have contributed enormously to our understanding of these conditions and are offering new insights, which we hope will be helpful for improved diagnosis and management of patients with these devastating disorders.

This review addresses the current concepts and advances in FTD.

## Clinical Features

### Behavioral variant (bv-FTD)

The clinical hallmark of bv-FTD is a disturbance in the personality and behavior, with changes of mood, motivation, and inhibition, leading to profound social disruption.[1,21,22] As the initial symptoms are neuropsychiatric, without impairment on cognitive screening tests, or overt changes on structural imaging,[23,24] these patients may be inappropriately diagnosed as suffering from a psychiatric disease, usually, depression or personality disorder.[20,25]

These changes become gradually evident to relatives, colleagues, and friends, because of disruption in their work performance, social, and family relationships. The effect on care is vivid with a high level of burden and stress.[26]

Patients may perform normally on standard neuropsychological tests of memory, language, attention, and visual spatial ability, but more recent tests designed to assess emotion processing,[27] social cognition,[28] theory of mind,[29] and complex decision making[30] are more sensitive and may show deficits in early cases, even if standard cognitive battery are normal.[24]

The most common features[31] of bv-FTD are shown in Table 1.

**Table 1:** Most common symptoms in bv-FTD[31]

Impaired Insight
Apathy
Disinhibition
Distractibility
Abnormal eating behavior
Stereotypic and ritualistic behavior
Impaired empathy
Mental rigidity
Dysexecutive symptoms
Speech adynamism

In order to simplify the clinical picture, this myriad of neuropsychiatric manifestations may be classified in three main groups

- Positive symptoms: These include disinhibition with lack of concern about social norms or embarrassment, impulsivity, outburst of violence, stereotypic and ritualistic behavior, abnormal appetite for sweets or gluttony, and impaired emotional judgment. When they are present, they strongly suggest the diagnosis of FTD.[22,24,32]
- Negative symptoms: These include apathy and inertia, emotional blunting, impaired insight, lack of interest in usual or leisure activities, decline in the amount of speech (adynamism or laconic speech), and reduce self-care for complex instrumental activities.[33] These symptoms are less specific to FTD and also occur in depression.
- Cognitive symptoms: These often appear later and include mental rigidity; loss of flexibility and abstraction; impairment in the pragmatic level of the discourse, with disorganization and distractibility; and poor planning and organization. At this stage, most patients fail in executive tasks and may show frontal release sign, such as grasping.[34,35]

This cluster of symptoms (positive, negative, and dysexecutive) have putative anatomical correlate to the orbitofrontal, medial, and dorsolateral frontal cortices, respectively.[33,36,37] Moreover, the progression of the atrophy, and consequently the clinical manifestations, may follow a predictable fashion, beginning in orbitofrontal and medial aspects of frontal cortex, and then involving the dorsolateral cortex and temporal anterior structures and basal ganglia.[38,39]

It has become increasingly apparent that some patients presenting with symptoms suggestive of bv-FTD fall to progress even over many years.[40] These “phenocopy” cases may actually constitute an unusual presentation of other conditions, such as late onset of bipolar disorder, personality disorder, or Asperger spectrum disorder. A number of features set apart these nonprogressive patients; normal performance on test of executive function[41] and of emotion processing;[27] better activities of daily living; and absence of brain atrophy.[40,42] The underlying pathophysiology in this group is unclear, but we assume that it reflects function disruption of orbitomesial frontal regions[43] in the absence of neurodegeneration and importantly a lack of atrophy on MRI[40] and hypometabolism on FDC-PET.[42] Table 2 displays characteristics that may distinguish one from another.

**Table 2:** Comparison between Bv-FTD and phenocopy

Feature	Bv-FTD	Phenocopy
Progression	+++	−
Atrophy on MRI	++	−
FDG-PET Changes	+++	−
Executive tasks impairment	++	+/−
Sleep disturbance	+++	−

### Semantic dementia

**Figure 1:**
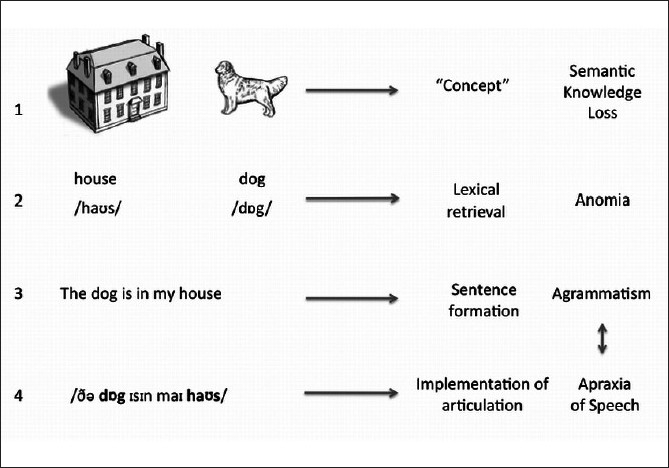
Stages of language production and PPA: In SD (1), there is a loss of semantic knowledge, so that the patient is not able to retrieve or comprehend words. In LPA (2), there are problems with word retrieval, but object recognition and word comprehension are intact. Whereas, in PNFA there is either disturbance in the word arrangement (agrammatism) (3), impairment in motor implementation of the speech (Apraxia of Speech) (4), or both (3 and 4).

Patients typically present with “loss of memory for words” and show impairment on tests of word comprehension, although the underlying deficit is the amodal store of semantic memory or knowledge about words, objects, people, and sounds.[7] [see Figure 1]. Patients show a gradual reduction of vocabulary and use high frequency terms (thing, boy), although speech is fluent and well articulated, without phonological or syntactic errors.[8,44,45]

A consistent feature is the impairment of naming objects or anomia. The performance is influenced by the level of familiarity and specificity of items asked. In other words, if the item is extensively encountered by the patient, it is likely to be forgotten later.[45] Likewise, the patient will tend to name objects that are prototypic of their category.[46] For instance, patients are able to name cat, dog, and horse, but not tiger or zebra, and use superordinate or general labels, calling the latter also a cat and horse, and may be just animal.[47]

Impairment of single word comprehension can be assessed by asking the patient to match the word with the corresponding object or to define the meaning of words.[45] There is a striking dissociation between repetition (preserved) and meaning (loss), best demonstrated by asking patients to repeat words such as “hippopotamus” or “catastrophe” and then to say what they mean.[48] These patients also show surface dyslexia where all words are read according to general rules of pronunciation, regardless of word meaning.[8]

In spite of the fact that word-based tests show clear deficits, actually there is a deterioration of central, amodal knowledge about objects or people which is apparent when nonverbal tests, such as “Pyramid and Palms test ” are employed, which requires subjects to match the two pictures that go together.[49]

Some SD patients present prominent deficits in identification of famous people. This deficit represents a general impairment on the “knowledge of people” than merely prosopagnosia (i.e., loss of ability to recognize faces), since patients are unable to produce any information from their name or voice.[50] Such patients typically show predominant right temporal atrophy[51] together with behavioral symptoms and poor insight.[52,53]

## Progressive Nonfluent Aphasia

Unlike SD, the presenting features of PNFA are more varied and may reflect breakdown at various stages of speech production, from alterations in lexical retrieval, misarrangements of the words according to grammatical rules, or impaired motor programming of the intended utterance.[11]

Generally speaking, there are problems with the syntactic or motor aspects of speech, causing speech to be halting, slow, and distorted.[54]

Severe agrammatism causes oversimplification of the language production, lack of function words (e.g., prepositions, auxiliary verbs, or articles), or words inflections (i.e., endings of verb or noun according to conjugation or number, respectively).[10] But in the early stages, grammatical errors are subtle and may be difficult to distinguish from common errors or detect in a short interview. Syntactic problems are usually best assessed by testing sentence comprehension.[55]

Breakdown in the motor programming is referred to as apraxia of speech, which causes distortion of output with pauses, speech errors, and loss of melody. Patients may have difficulty in repeating or pronouncing polysyllabic words and strings of syllables (such as Pa-Ta-Ka), producing distortions and aprosodic intonation.[56]

Orobuccal apraxia may develop and some patients evolve into a picture of CBD or PSP.[57]

### A third language variant: Logopenic/phonological

Gorno-Tempini *et al*. described cases of language variant that fulfilled neither SD nor PFNA criteria.[58] These cases showed reduced speech output with frequent pauses and impaired naming; preservation of grammar, motor speech, and semantic knowledge. A remarkable feature was profound impairment on repetition of sentences or string of words and difficulties in understanding complex instructions, despite of sparing single word repetition and comprehension. This has been attributed to a reduction of working memory resources, due to impairment of phonological loop.[59]

Interesting enough, this group showed a distinctive pattern of brain atrophy that involved the left temporoparietal junction. There is growing evidence that the underlying pathology is Alzheimer’s disease, suggesting that this variant is in fact, an atypical presentation of AD.[58,60,61]

Table 3contrasts the clinical features of the three language variants and Figure 1 shows the stages of language production involved.

**Table 3:** Clinical features of language variants

Feature	SD	PNFA	LPA
Agrammatism	−	+++/− *	−
Motor speech disorder	−	+++/− *	−
Anomia	+++	+	+++
Single word comprehension	+++	−	−
Comprehension complex or sequential instructions	−	++	+++
Single word-repetition	−	++	−
Sentence repetition	−	++	+++
Surface dyslexia	+++	−	−

* Either agrammatism or motor speech disorder must be included.

### Imaging

The advent of high resolution MRI and of methods of automated qualification such as Voxel-based morphometry (VBM)[62] and cortical thickness measures[63] has enhanced our knowledge of the anatomical changes in the variants of FTD.

Patients with bv-FTD show atrophy of the orbitobasal and medialfrontal lobes, together with anterior temporal and insular involvement.[39,64] SD is associated with atrophy of the anterior temporal lobe involving particularly polar, anterior parahippocampal, and fusiform regions including the perirhinal cortex. The atrophy is bilateral, but typically asymmetric and often more severe on the left.[58,65] In PNFA, the changes are subtler and involve the left inferior frontal lobe and anterior insula cortex.[58,66,67] In logopenic/phonological variant the atrophy involves the left hemisphere, particularly the posterior temporal lobe (superior and middle temporal gyri) and inferior parietal lobe and lesser involvement of the precuneus.[58,59,68]

These changes can also be detected using simpler MRI-based visual rating scales, which simply use standard coronal cuts. These scales aid diagnosis and monitoring of progression.[69]

### Pathology

Definitive diagnosis of FTD requires neuropathological examination. Unlike other dementia syndromes, notably AD, FTD encompasses considerable pathological heterogeneity.[2,18,70,71] The classification is based on the identification of intracellular protein inclusions by means of inmunohistochemistry.[71] Accordingly, three broad subdivisions have been recognized:

FTD with tau-positive inclusions: This includes classic Pick’s disease, Progressive Supranuclear Palsy, Corticobasal degeneration, argyrophilic grain disease, and patients with mutation of the Microtubule-associated protein tau (*MAPT*) gene on chromosome 17 (FTDP-17).[71]

FTD with tau-negative, ubiquitin-positive inclusions: This is the commonest pathological finding in FTD[70,72,73] and includes these with progranulin gene mutations.[74] The ubiquitinated protein has been identified as the *transactive response DNA-binding protein with Mr* 43 (TDP-43) which is also found in MND,[75,76] strengthening the association between FTD and MND. Interestingly, cases with ubiquitinated lesions without TDP-43[77] have been recently identified, which appear to have abnormal deposits of another protein called *fused in sarcoma protein* (FUS).[78]

Dementia lacking distinctive histology. Includes cases that do not show any particular or distinctive inclusion or histology, besides neuronal loss, superficial spongiosis, and gliosis. With the advent of newer inmunohistochemical techniques such cases are now increasingly rare.[71]

**Figure 2:**
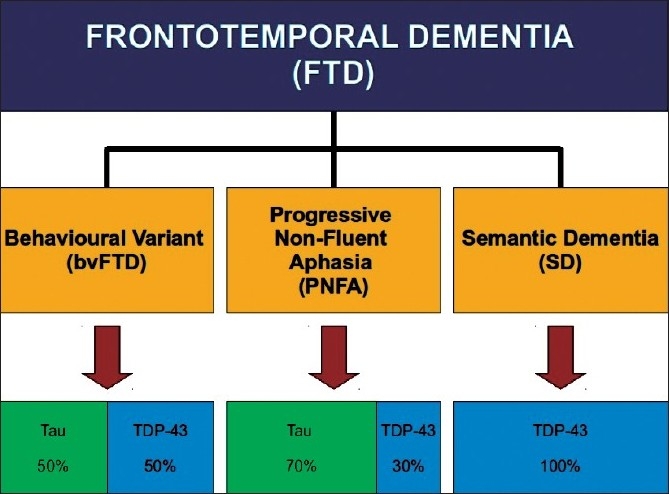
FTD syndromes and pathology

The correspondence between clinical phenotype and underlying pathological subtype has been a topic of considerable interest in recent years.[18,70,79] As shown in Figure 2, SD has been consistently associated with Ubiquitin (TDP-43) positive pathology.[9,72,80,81] In contrast, PNFA cases show variable results in different clinicopathology series, due probably to different diagnostic criteria employed as well as inclusion of logopenic cases in older series.[9,18,61,70,73,82] Despite that, PNFA more often associated to tau pathology, particularly if there is motor speech disorder.[83] In bv-FTD approximately a half has tau-positive and the other TDP-43-positive pathology.

### Genetic

Around 40% of patients report a family history of dementia, although in many instances this is almost certainly unrelated, but 10–20% have a clear pattern of autosomal dominant inheritance, with at least two relatives having young onset dementia or MND.[14,84,85] The heritability, however, varies according to the variant FTD: SD showing the least, whereas bv-FTD and FTD with MND the most inheritable.[14,81]

The commonest identified mutations are *MAPT* and *progranuline* (*PGRN*), both in chromosome 17q21.[86,87] Although the prevalence of mutations varies among studies, the two mutations have a similar frequency, being found in around 5–10% of patients.[88] Other mutations involve the Valosin-containing protein (VCP) and CHMP2B genes, but are very rare.[89]

The mutations of *MAPT* lead to abnormal intracellular accumulation of hyperphosphorylated tau.[90] While mutation of *PGRN* gene results in reduced expression of progranulin and is associated with Ubiquitin-TDP-43 pathology.[91] *MAPT* mutations typically give rise to the clinical phenotype called Frontotemporal dementia and parkinsonism linked to Chromosome 17 (FTDP-17). As its name suggests, the clinical picture embraces isolated behavioral and personality changes, initial extrapyramidal signs, suggesting of PSP or CBD, or a combination of behavioral and extrapyramidal syndromes.[92] In contrast, *PGRN* mutations appear to produce striking clinical heterogeneity which includes CBD, PNFA, or bv-FTD. Moreover, some cases depict an anmestic syndrome compatible with initial AD and a logopenic aphasia.[91]

### Future directions

In the last twenty years, a great deal of progress on molecular genetic and imaging has led to new insights about FTD syndromes. New imaging methods, for instance VBM, has given a detailed account of pattern of brain atrophy, allowing an unbiased comparison of patients groups, while the development of radiotracers, such as PiB has enabled to identify the accumulation of extracellular beta-amyloid, and therefore, rule out cases of AD. Ligands specific to tau and TDP-43 are eagerly awaited.

Advances in neuropsychological assessment have also led a better understanding of the language and social cognitive difficulties seen in FTD.

Many issues remain unresolved. The relationship between genetic, pathologic, and clinical phenotype is of key importance as is the ability to identify pathological subtypes in vivo by the use of biomarkers. Eventually, it is hoped that biomarkers will be identified so that a specific therapy can be tailored according to the underlying pathology. The degree of overlap between MND and FTD syndromes is also a topic of keen current research interest.
